# Executive Summary of the Assessing Surgical Site Infection Surveillance Technologies (ASSIST) Project

**DOI:** 10.1089/sur.2019.171

**Published:** 2019-09-10

**Authors:** Heather L. Evans

**Affiliations:** Department of Surgery, Medical University of South Carolina, Charleston, South Carolina.

**Keywords:** surgical wound infection, mobile health, patient generated health data, smartphone, postoperative care, technology assessment

## Abstract

***Background:*** The expert panel that conducted the Assessing Surgical Site Infection Surveillance Technologies (ASSIST) project elaborates on the key findings of the health technologies assessment (HTA) report in a series of articles addressing topics from workflow challenges to implementation strategies to new big data analytics tailored to incorporate serial patient-generated health data (PGHD).

***Conclusion:*** By reporting on the methodology, with an emphasis on stakeholder engagement, the ASSIST investigators provide the basis for a future deep dive into the next phase of PGHD integration into surgical site infection (SSI) surveillance.

The adoption of smartphones, texting, and patient portals for post-operative care coordination present both challenges and opportunities to the surgeon. Real-time communications that were once the fantasy of Dick Tracy and *Star Trek* are now second nature to digital natives, who bring their own devices to healthcare with the expectation that providers will review patient-generated health data (PGHD) and engage via new communication channels. It is now possible to offer expanded, personalized care to patients after surgery, using new data streams and data types via mobile devices to facilitate remote patient monitoring. One of the most compelling use cases for post-operative mobile health (mHealth) tracking is the triage of surgical sites for evidence of surgical site infection (SSI) through review of serial incision photography and symptom reporting.

Although the current standard of care for SSI diagnosis requires in-person physical examination of the surgical site, patient-generated photographs are increasingly submitted to and reviewed by surgical providers via e-mail, text messaging, and electronic health record-based patient portals. Although the current use of telemedicine for post-discharge surgical care has been reviewed systematically, the specific use of mHealth for SSI surveillance and the process of work associated with this activity is highly variable and generally unacknowledged in the medical literature. In September 2017, the Safety and Healthcare Epidemiology Prevention Research Development (SHEPheRD) program [1] from the U.S. Centers for Disease Control and Prevention (CDC) awarded the University of Washington the opportunity to conduct the Assessing Surgical Site Infection Surveillance Technologies (ASSIST) project aimed at the evaluation of the current use of PGHD and mobile devices in post-operative SSI surveillance. The purpose of the project was to conduct a health technology assessment (HTA) of the state of the science of using mHealth for SSI care, and to make recommendations to the CDC for further work to facilitate the integration of PGHD into the standards for SSI detection and surveillance.

In year one, the ASSIST investigators completed a literature review and a landscape analysis of apps directed specifically at post-operative incision monitoring. Through this initial work, the group also developed a network of stakeholders (researchers, patients, clinicians, administrators, and health information technologists among them) and conducted key informant interviews to gain additional perspective on real-world use. In May 2018, the group hosted a one-day Patient-Generated Health Data Stakeholder Advisory Group workshop to seek feedback from key opinion leaders on the findings from the HTA process. A report from this workshop is available online at www.cirg.washington.edu/assistPSAGreport [2]. At the beginning of year two, the ASSIST group met and assembled 10 recommendations drawing from stakeholder discussions to address remaining gaps in knowledge, advise best practice in the application of mHealth for SSI surveillance (listed in Table 1). A draft report was completed in January 2019 and disseminated online for public comment, and the final version of the report submitted to the CDC in May 2019 [3].

**Table 1.** Recommendations from the ASSIST Project

1. Tools and programs that use PGHD captured via mHealth for SSI surveillance should be designed and implemented with direct involvement of core beneficiaries and stakeholders. This includes patients, providers, and administrative staff who are the primary users of mHealth tools and programs.
2. Best practices and standards for privacy and security of PGHD captured via mHealth for SSI surveillance should be established and followed rigorously. Protection of PGHD presents unique challenges because of its nature as data originating outside of the healthcare environment, and as data that “belongs” to patients.
3. Design of tools and programs that use PGHD captured via mHealth for SSI surveillance should address the new complexities presented to workflow, IT integration, and communication. This includes provider and administrative staff workflows inside and outside the clinic, as well as integration with existing health IT infrastructures.
4. Design of tools and programs that use PGHD captured via mHealth for SSI surveillance should acknowledge and account for the work performed by patients outside the healthcare setting. Collection and reporting of patient-generated health data by patients who are most likely in a post-surgical state entails additional burdens on time and energy.
5. To expedite the generation of evidence for using PGHD captured via mHealth for SSI surveillance, a Community of Practice should be established, including participation from the full range of stakeholders. This Community of Practice would continue collaboration to identity valuable activities to advance knowledge and practice, support efficient dissemination of research results, support the development of methods for the implementation of PGHD captured via mHealth for SSI surveillance, and enable practitioners and researchers to draw on the knowledge and experience of leaders in the field.
6. For continued advancement of PGHD captured via mHealth for SSI surveillance, researchers and health systems should look to other disciplines and non-surgical specialties where technology and programs for mHealth and PGHD are in a more advanced state, including in tele-dermatology, burn care, and chronic wound care.
7. Research on PGHD captured via mHealth for SSI surveillance should include the development of a database of patient-generated post-operative incision photos. Such a database would make available for research a robust data set for the examination of post-operative incision health and the range of post-operative incision appearance.
8. Data generated through PGHD captured via mHealth for SSI surveillance should be leveraged to characterize the natural history of SSI better and inform a review of current clinical and public health practices and surveillance standards for identifying and diagnosing SSI.
9. Implementation science frameworks address program sustainability, scalability, and replicability, and increase the likelihood of success of future programs. Implementation of science frameworks and methods should guide the deployment and evaluation of programs that utilize PGHD captured via mHealth for SSI surveillance to ensure equitable health care access and cost arrangements.
10. Metrics used to assess core outcomes of PGHD captured via mHealth for SSI surveillance should align with value propositions held by stakeholders, including patients, providers, administrators, payers, researchers, and public health. Such metrics include patient satisfaction/experience, health outcomes, healthcare utilization, and public health data utilization.

Assessing Surgical Site Infection Surveillance Technologies; mHealth, mobile health; PGHD = patient-generated health data; SSI = surgical site infection; IT = information technology.

In this special issue of *Surgical Infections*, the expert panel that conducted the ASSIST project elaborates on the key findings of the HTA report in a series of articles addressing topics from workflow challenges to implementation strategies to new big data analytics tailored to incorporate serial PGHD. Acknowledging the rapid development cycle used in design and deployment of mHealth apps, the ASSIST investigators concede that any report from a defined period of time can best be regarded as a biopsy, as the field continues to grow unabated. Even since the conclusion of the project, new clinical trials of mHealth apps for post-operative care have been presented [4] and published [5], and a current study in Europe focusing on time to SSI detection should complete enrollment this summer [6].

The number of mHealth apps increases exponentially each year, and as new apps emerge, older ones may mature and expand or regress into the past with little usage or impact on patient care. Furthermore, some of the apps reviewed may never advance to commercialization, remaining research endeavors for their whole lifespan. But these orphan apps have value, in that the process of development, the features contained, and the implementation trials all frame lessons for future app design, integration and dissemination. Additionally, the data collected can serve as fuel to drive innovation in analytic methods and to incorporate PGHD, especially incision images, into the clinical and surveillance definition standards for SSI. Finally, by reporting on the methodology, with an emphasis on stakeholder engagement, the ASSIST investigators provide the basis for a future deep dive into the next phase of PGHD integration into SSI surveillance.

## The ASSIST Investigators

Heather Evans, Medical University of South Carolina; William Lober, University of Washington; Danielle Lavallee, University of Washington; Sheri Chernetsky Tejedor, Emory University, Centers for Disease Control; E. Patchen Dellinger, University of Washington; Traci Hedrick, University of Virginia, Shuai Huang, University of Washington; Dan Pollock, Centers for Disease Control and Prevention; Aven Samareh, University of Washington; Robert G. Sawyer, Western Michigan University; John Semple, University of Toranto; Jyotirmay Sharma, Emory University; Elizabeth Wick, University of California San Francisco; Jenney Lee, University of Washington; Sarah Lawrence, University of Washington.
